# Exploring Audiologic Outcomes in Sudden Sensorineural Hearing Loss: A Retrospective Analysis

**DOI:** 10.7759/cureus.52977

**Published:** 2024-01-26

**Authors:** Cecilia Belen Espinosa-Arce, Leon Felipe I Garcia-Lara, Martha L Martinez-Servin, Antonio R Villa, L Stefano Ramirez-Gil

**Affiliations:** 1 Department of Otolaryngology–Head and Neck Surgery, Hospital Angeles Metropolitano, Mexico, MEX; 2 Department of Otolaryngology–Head and Neck Surgery, Hospital Central Sur Petróleos Mexicanos, Mexico, MEX; 3 Department of Otolaryngology–Head and Neck Surgery, Instituto Mexicano de Otologia y Neurotologia, Mexico, MEX; 4 Department of Audiology and Otoneurology, Hospital Central Sur Petróleos Mexicanos, Mexico, MEX; 5 Department of Investigation, Faculty of Medicine, Universidad Nacional Autonoma de Mexico, Mexico, MEX

**Keywords:** treatment outcome, audiometry, salvage therapy, adjuvant treatment, sudden sensorineural hearing loss, intratympanic steroid injection

## Abstract

Objective

This retrospective study aims to present the audiologic outcomes of patients aged 18 years and above who underwent treatment for sudden sensorineural hearing loss (SSNHL) at the tertiary Hospital Central Sur Petróleos Mexicanos in Mexico City, Mexico, between January 2000 and December 2015.

Main outcome measures

The main outcome measures were patient demographics (age, sex, comorbidities) time from symptom onset to diagnosis and treatment initiation, initial threshold, treatment details (type, dosage, duration), adverse effects, audiometry at diagnosis and at the end of treatment, follow-up duration, and pure-tone average.

Results

A total of 72 patients were included, with a mean follow-up duration of four months. Comorbidities such as type 2 diabetes mellitus, hypertension, and hypertriglyceridemia were observed in a significant portion of patients. However, these conditions and the use of salvage therapy and adjuvant drugs did not impact hearing recovery. A longer delay from symptom onset to medical attention was associated with a lower gain in decibels (p=0.307). Diabetic patients who received steroid treatment showed a significant gain of at least 15 dB, indicating the greatest benefit in this subgroup.

Conclusions

Adjuvant drugs may be unnecessary and ineffective in treating SSNHL. Metabolic disorders may be linked to the development of SSNHL. Steroid treatment is the only effective therapeutic option for improving hearing recovery in diabetic patients. Early initiation of treatment after symptom onset is crucial for maximizing auditory recovery.

## Introduction

Sudden sensorineural hearing loss (SSNHL) is a significant otologic emergency, with the majority of cases being idiopathic in nature. The National Institute of Deafness and Other Communication Disorders (NIDCD) defines it as a sudden sensorineural hearing loss of at least 30 dB over at least three consecutive test frequencies occurring within a 72-hour period [1].

The global incidence ranges from five to 20 cases per 100,000 persons per year [2]. This condition not only impairs patients' ability to engage in social interactions but also poses a risk to their safety by limiting their ability to hear important alert sounds, such as car horns, screams, and alarms. Additionally, it disrupts sound localization by affecting binaurality in cases of unilateral hearing loss or total hearing loss in patients with only one functional ear. Although SSNHL can affect individuals of any age, it is most commonly observed in the age range of 43-53 years [3].

Spontaneous recovery has been reported within the first three months, in the range of 32%-65% [4-6]. Many treatment options have been proposed and include systemic and topical steroids, antiviral agents, hyperbaric oxygen therapy (HBOT), diuretics, herbal and other complementary and alternative treatments, middle ear surgery for fistula repair, and observation alone [1].

The variety of treatment choices reflects the complexity of managing SSNHL. Patients and clinicians are often confronted with the challenge of selecting the most appropriate treatment option given the available evidence and individual patient characteristics.

In this study, we aim to contribute to the understanding of SSNHL treatment outcomes by investigating the audiological results of patients who received different treatment modalities, evaluate the time elapsed from symptom onset to the initiation of pharmacological treatment, and examine the comorbidities associated with this condition.

## Materials and methods

Study design, population, and outcome measures 

This study was approved by the local ethics committee. Informed consent is not necessary because of the retrospective nature of this study. The information was collected from the electronic records of patients over 18 years of age diagnosed with SSNHL using the ICD-10 code H912 from January 01, 2000, to December 31, 2015, of the tertiary hospital Central Sur Petróleos Mexicanos, Mexico. Exclusion criteria included patients without tonal audiometry at the time of diagnosis or at the end of treatment, patients with ruled-out diagnosis during follow-up, unspecified treatment details, and the lack of follow-up care in the unit.

Patient demographics (age; sex; comorbidities such as type 2 diabetes mellitus, systemic arterial hypertension, and dyslipidemia; time in days from symptom onset to diagnosis; time in days from diagnosis to treatment initiation; threshold at the time of diagnosis; and record of tonal audiometry results per patient) were recorded. The type of treatment indicated, treatment dosage, treatment duration, adverse effects presented, tonal audiometry at the end of treatment, and follow-up in weeks were also documented. Once the audiometric data were obtained, the pure-tone average (PTA) was calculated by averaging the thresholds at the frequencies of 500, 1,000, and 2,000 Hz, and the percentage of logoaudiometry was recorded in the database.

**Figure 1:**
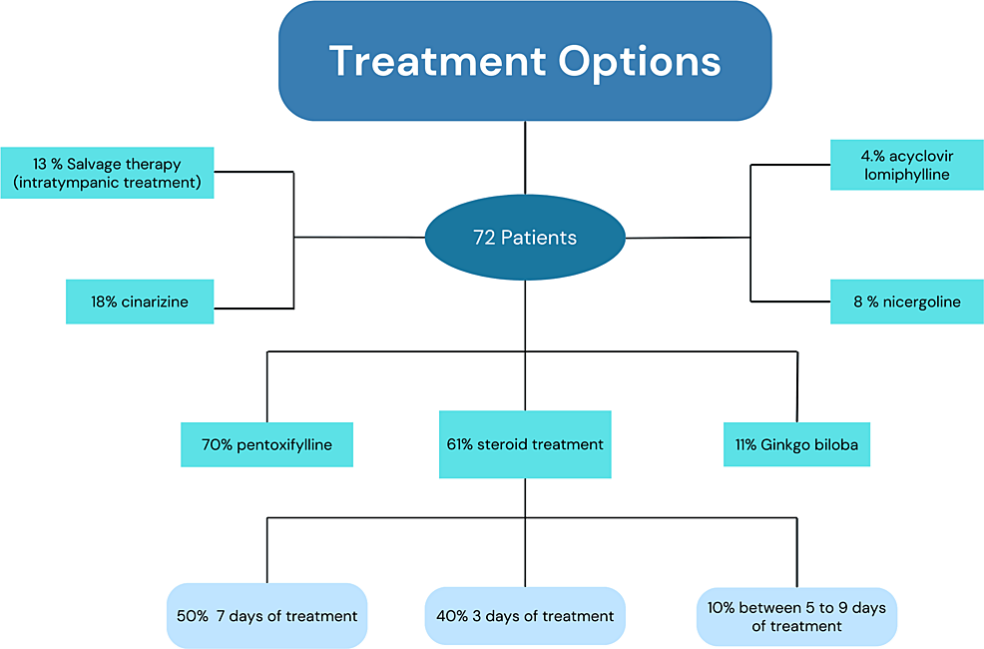
Flowchart showing the treatment options The figure shows that 61% of the sample (N=72) received oral steroids. Among them, half followed a seven-day regimen with this dose, 40% received it for three days, and the remaining 10% followed other regimens that included five and nine days. As salvage therapy, intratympanic treatment was used in 13% of the cases. Among other drugs, the patients also received: pentoxifylline in 70% of cases, cinnarizine in 18%, nicergoline in 8%, ginkgo biloba in 11%, acyclovir in 4.3%, and lomiphylline in 4%.

Figure 1 shows the treatment distribution. Patients received oral steroids at a maximum dose of 1 mg/kg/day, the maximum dose of steroids was 70 mg/day in 66% of the patients, while 13% received 60 mg/day. The regimen was gradually decreased over a total of 21 days in 47.7% of the patients and 30 days in 43%. Ten percent of the patients received a single dose of intramuscular steroid (betamethasone). As salvage therapy, intratympanic treatment was used with the following regimen: dexamethasone 8 mg/2 mL until the tympanic cavity was filled, with daily application for five days. Among other drugs, the only one used as monotherapy was pentoxifylline, in 9% of the patients, which corresponds to 12 diabetic patients.

The variable defined as "Gain in decibels," obtained from the difference between the diagnostic threshold and the control threshold, was divided into six groups for easier statistical analysis as follows: Group I: <0 dB, meaning negative numbers or zero; Group II: 5-9.9 dB; Group III: 10-19.9 dB; Group IV: 20-29.9 dB; Group V: 30-39.9 dB; and Group VI: greater than 40 dB. To analyze if the degree of hearing loss is relevant to the gain in decibels, the threshold value at the time of diagnosis was subdivided into four groups: diagnosis threshold for mild hearing loss: 20-39 dB; diagnosis threshold for moderate hearing loss: 40-59 dB; diagnosis threshold for severe hearing loss: 60-79 dB; and diagnosis threshold for profound hearing loss and beyond: >80 dB.

Statistical analysis

We performed several statistical analyses to evaluate the data. Measures of central tendency, such as the mean, median, and averages, were calculated to provide a summary of the data. To assess the differences in audiometric results before and after pharmacological treatment, we employed Student's t-test. P<0.05 was considered statistically significant.

## Results

**Figure 2:**
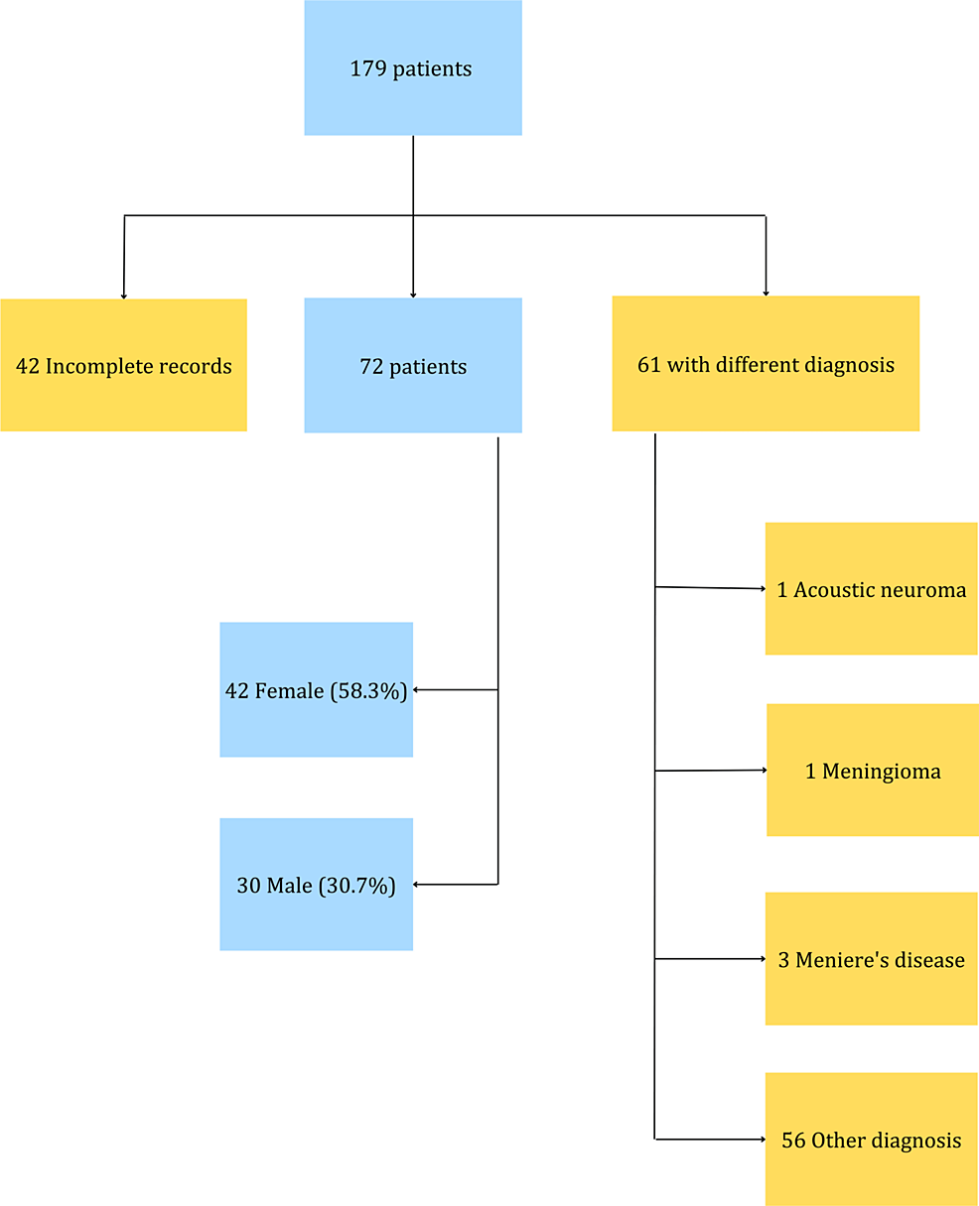
Flowchart showing patient distribution and final sample after exclusion criteria

In the initial search in the electronic medical records from January 1, 2000, to December 31, 2015, with the ICD-10 diagnosis H912 corresponding to SSNHL in both the audiology and otorhinolaryngology departments, a total of 179 patients were identified. Out of these, 72 cases were analyzed, as 42 did not have complete medical records, and 61 patients were misclassified with the diagnosis of idiopathic SSNHL. Among these cases, we excluded those who were initially treated as idiopathic but later found to have an etiology for the hearing loss: one meningioma, one Schwannoma, and three cases classified as Meniere's disease (Figure 2).

The age range was from 38 to 85 years, with a mean age of 60. The majority of the patients were female (58.3%), and the left ear was predominantly affected (65%). Only 25% had a history of upper respiratory tract infection prior to the onset of SSNHL. None of the patients had a history of gastrointestinal tract infection. Incidentally, one out of 10 patients reported detecting the onset of symptoms (hearing loss) after air travel. In addition to the main symptom of hearing loss, 22% of the patients reported vertigo, and 57% reported tinnitus.

Thirty percent of the study population had a diagnosis of type 2 diabetes mellitus, with the majority having a disease duration of less than 10 years (67%). Almost two-thirds (61%) were hypertensive, of which the majority (86%) had a disease duration of less than 10 years. Pre-existing diagnosis of dyslipidemia was present in 30% of the patients. Half of the population had serum triglyceride levels above 150 mg/dL and total cholesterol levels above 200 mg/dl during the episode of SSNHL. Among the total patients, 30% had a previously established diagnosis of dyslipidemia.

**Figure 3:**
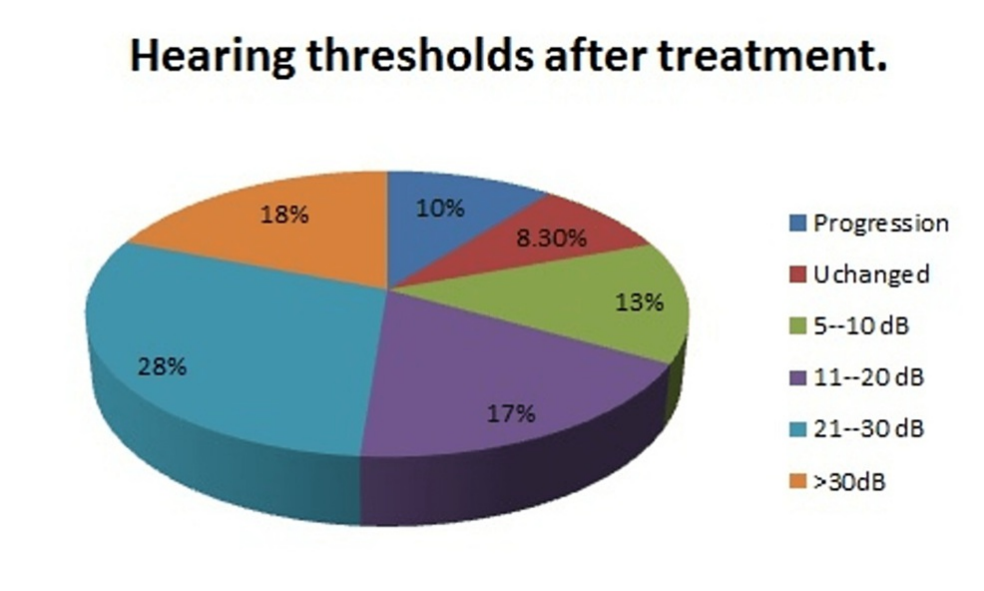
Hearing improvement after treatment The graph represents the distribution percentage of improvement in dB in comparison to the initial values.

The duration of follow-up varied among patients, with some having annual evaluations for up to a decade. In contrast, there were patients who only had one follow-up visit, in addition to the diagnostic assessment. On average, the follow-up duration for the case series was four months. The average hearing threshold obtained from the frequencies of 500 Hz, 1,000 Hz, and 2,000 Hz was mostly above 50 dB (60%), indicating moderate to severe hearing loss. The follow-up studies showed an overall improvement in hearing thresholds; as in 60% of the cases, the thresholds were lower than 50 dB. Ten percent of the study population showed progression of hearing loss, reflected in an increase in the auditory threshold in the follow-up study. Moreover, 8.3% remained unchanged, 13% showed improvement between 5 and 10 dB, 17% between 11 and 20 dB, 28% between 21 and 30 dB, and 18% above 30 dB, indicating that 80% of the patients had some degree of auditory improvement (Figure 3).

The time elapsed between the onset of symptoms and the first specialized medical attention occurred within the first 24 hours in 20% of the cases. Thirty-seven percent received attention between days two and seven, 23% sought medical care within one month, 10% between one and three months, and there were four cases where patients sought consultation for the first time after one year of symptom onset. Within the diagnostic evaluation of the patients, a cranial tomographic study was requested in 23.6% of cases, with no documented abnormalities explaining sudden hearing loss in any case. A cranial magnetic resonance imaging (MRI) was performed in 37% of the cases, with abnormal results found in 5% (ischemia in one case and pontine bulb angioma in another case). In 15% of the cases, Brainstem auditory evoked potentials (BAEPs) were requested, and they were abnormal in one case, which was excluded due to the diagnosis of vestibular schwannoma.

When performing a cross-analysis between patients with tinnitus and the gain in decibels, the values in the gain in decibels did not have a relationship with the presence of tinnitus (p=0.183). The same analysis was performed for vertigo and gain in decibels, without finding a correlation (p=0.45). The presence or absence of any of the following factors did not make a difference among the groups of auditory gain in decibels: gender and age (cut-off point: 60 years as the mean). There was an inversely proportional relationship, with younger age being associated with greater gain in decibels (p>0.05). However, up to 40% of individuals under 60 years had gains greater than 30 dB, while 40% of those over 60 years had either deterioration or gain less than 10 dB, type 2 diabetes mellitus, systemic arterial hypertension, treatment with oral steroids at 1 mg/kg/day, days of maximum dose with systemic steroids, total days of systemic steroid treatment, treatment with intratympanic steroids (as salvage therapy), treatment with pentoxifylline, and treatment with Ginkgo biloba.

**Figure 4:**
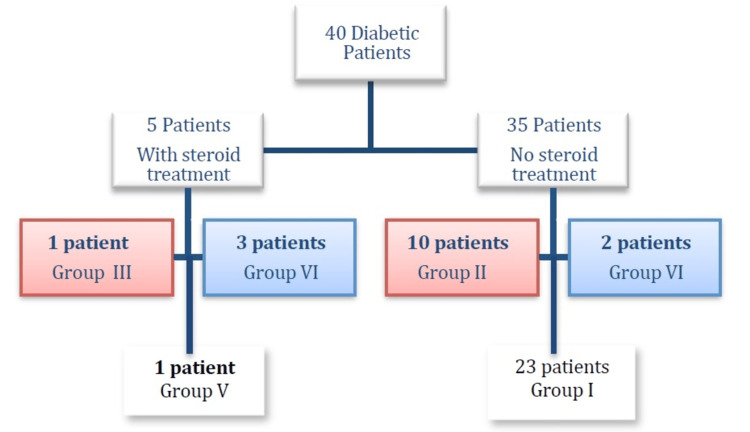
Treatment and hearing results of diabetic patients Forty patients of the sample were diabetic, and only five received steroid treatment that led to greater hearing improvement in comparison with the 35 patients who did not receive steroids; the gain in decibels were classified into six groups; the higher the group, the greater the improvement (p<0.05).

When analyzing the cases of diabetic patients, the following observations were made: 29% of the sample were diabetic, and out of 40 diabetic patients, only five were prescribed oral systemic steroids at a maximum dose of 1 mg/kg/day. Among these five cases, three were classified in Group VI, meaning patients with a gain in decibels greater than 40 dB. One was classified in Group V (gain between 30 and 39.9 dB). The fifth patient was classified in Group III; in summary, all of them had a gain of at least 15 dB. None of the diabetic patients who received steroids experienced a threshold deterioration. Among the diabetic patients who did not receive steroids, the results in terms of auditory gain were highly variable. Only two patients had a gain greater than 40 dB. Ten patients had minimal gain, placing them in Group II with the rest remaining unchanged in Group I (Figure 4).

**Figure 5:**
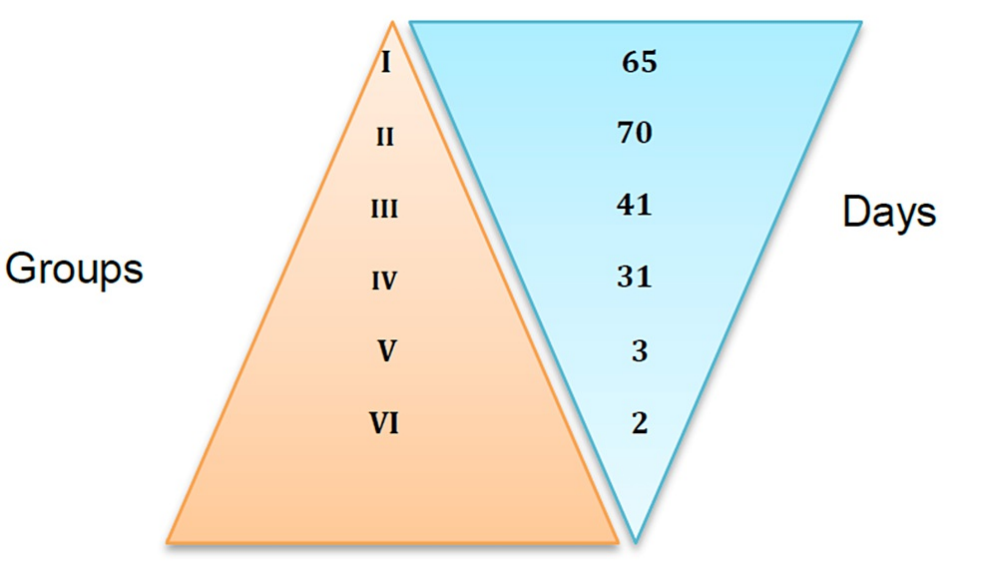
Relationship between gain in decibels and elapsed days from the onset of symptoms to medical attention The gain in decibels was classified into six groups; the higher the group, the greater the improvement. Patients that received early treatment showed better results in terms of decibel gains with the greatest effect being seen in the first seven days.

The mean number of days elapsed from the onset of symptoms to medical attention in relation to auditory gain in decibels was as follows: Group I, 65.7 days; Group II, 70.8 days; Group III, 41.93 days; Group IV, 31.89 days; Group V, 3.57 days; and Group VI, 2.88 days. There was an inverse relationship between the gain in decibels and the number of days elapsed from the onset of symptoms to medical attention. However, when conducting an ANOVA analysis, a p-value of 0.307 was obtained (Figure 5).

A cross-analysis was performed between the groups of decibel improvement and diagnosis threshold, and no difference was found (p>0.05). However, no clear trend was observed. The number of cases with a gain in decibels in Group VI is the same as in Group I or II, despite having different diagnosis thresholds.

The presence of phonemic regression curves in the speech audiometry was detected in 18.2% of cases at the time of diagnosis, and only in 11% in the last study conducted. Among the 35 patients treated with oral steroids, seven had phonemic regression curves at the time of diagnosis, of which five cases showed the disappearance of this curve in the final control. On the other hand, in three cases, there was no phonemic regression curve at diagnosis, but it appeared in the posterior study. Each case was reviewed individually, and it was found that the presence of phonemic regression was not recorded at the beginning because phonemic discrimination was absent. Iit can be assumed that there was some degree of phonemic discrimination after treatment.

## Discussion

Comorbity association 

Our findings indicate that 50% of patients with SSNHL exhibit abnormal cholesterol levels and hypertriglyceridemia. The association between SSNHL and comorbidities has been investigated in several studies. A case-control study found that the 'metabolic syndrome' was identified as a significant risk factor for SSNHL with an adjusted odds ratio of 4.30 (95% CI: 1.98-9.33; p<0.001) [7]. Similarly, a systematic review and meta-analysis revealed that the prevalence of diabetes and hypertension was higher among patients with SSNHL compared to control groups. The odds ratio for diabetes was 1.61 (95% CI: 1.31, 1.99). For hypertension, the odds ratio was 1.50 (95% CI: 1.16, 1.94). Furthermore, cholesterol levels were higher in patients with SSNHL compared to controls, but no significant associations were found for smoking, HDL, LDL, triglyceride, or BMI [8]. In contrast, two studies found no association between SSNHL and comorbidities such as hypertension and diabetes, as well as smoking and drinking habits [9,10] or hyperlipidemia [10]. Alternatively, one study found higher levels of leukocytes, neutrophils, neutrophil-to-lymphocyte ratio, and fibrinogen in patients with SSNHL [9].

The various comorbidities discussed earlier highlight the complexity of the disease, with no clear single cause identified, but rather a combination of interactions. However, it indicates a possible connection between metabolic disorders, hypercoagulability, inner ear vascular microthrombosis, and the occurrence of SSNHL.

Prognostic factors 

Usually, the literature mentions various factors related to poor outcomes. These include vertigo [11], delayed treatment [11,12], the presence of metabolic syndrome [11,13], age [12], and severity of initial hearing loss [7,13,14]. However, a case-control study by Chien et al. found that hearing loss severity was not a significant predictive outcome factor [11]. A retrospective study in profound SSNHL found no specific factors important for hearing loss recovery (age, gender, BMI, hypertension, diabetes) [14]. Another retrospective study found that patients with SSNHL and diabetes mellitus had a higher initial hearing threshold and were hospitalized for a longer time. However, it did not differ in hearing recovery in comparison to non-diabetic patients [15].

In our study, we did not identify any specific factors related to the prognosis or treatment response, except for the negative impact of delayed treatment on auditory gain in decibels. Two studies [3,16] emphasize the importance of early treatment within seven days of symptom onset, as corticosteroid treatment seems to yield the best recovery outcomes, particularly in the first two weeks. Early treatment stands out as the most crucial prognostic factor for hearing recovery.

Steroid treatment

We found no difference in the use of one medication over another in the final hearing recovery.

Steroid treatment has been the primary approach for SSNHL but with mixed results regarding its effectiveness. Studies have demonstrated positive effects on hearing improvement [17], while others have not found it to be superior to placebo [18,19]. Though the balance of benefit versus harm is uncertain, there is insufficient evidence to say it is ineffective. Offering this treatment to patients is reasonable due to the potential for hearing improvement.

The only case in our study where there is evidence favoring steroid treatment is in diabetic patients, despite being advised against it, showed the most benefit from its use in our study. We observed only one case of complications from steroid side effects, indicating the treatment to be safe. However to avoid systemic corticosteroid side effects, intratympanic administration should be preferred, as it shows no significant difference in hearing recovery [3,20]. 

Salvage therapy

In our study, the use of salvage therapy with IT steroids did not show significant differences in auditory gain in decibels among the groups. Previous studies have demonstrated that IT salvage treatment with steroids can lead to significant improvement in hearing recovery [21,22], particularly in patients with total deafness [22]. However, the certainty of evidence is considered low to moderate due to potential bias.

Two randomized controlled trials reported hearing improvement in 37%-48% of patients who received salvage IT steroid treatment [23,24].

A systematic review and meta-analysis comparing HBOT and IT salvage therapy as independent treatments found no significant difference in PTA changes (p=0.64) [25]. In a randomized trial, PTA improvement was observed, particularly in patients who received a combination of HBOT and oral steroids (p<0.05). Patients treated with HBOT showed better recovery compared to those treated with oral steroids alone (p<0.05). The timing of treatment initiation also played a role, with patients who started therapy within seven days from SSNHL onset showing significant PTAv recovery when treated with HBOT or HBOT + oral steroids (p<0.05), but not with oral steroids alone (p=0.08). For patients treated more than 14 days after the SSNHL onset, the combination of HBOT and oral steroids resulted in better PTA recovery compared to HBOT or oral steroids alone (ANOVA: p=0.08) [26]. Another systematic review and meta-analysis also demonstrated significant improvement in final PTA and hearing recovery with the combination therapy of steroids and HBOT [27].

The lack of positive effects observed in our study with IT salvage treatment could be attributed to two factors: the treatment was used alone without combining it with HBOT, and there was a significant delay in initiating treatment after the SSNHL onset.

Adjuvant treatments 

In our study, we find adjuvant treatments to be ineffective in hearing recovery. Studies investigating the use of Ginkgo biloba as an adjuvant medication have shown some positive effects in speech discrimination [28], clinical cure rate, total effective rate, and PTA [29] compared to steroid treatment alone. However, the evidence quality was inconsistent and was rated low to very low, suggesting a potential risk of bias. Additionally, a retrospective study in diabetic patients found no significant difference in PTA improvement and recovery between steroid treatment and pentoxifylline treatment (p>0.776), with a higher occurrence of hyperglycemic events in the steroid group (p=0.044) [30]. To draw definitive conclusions and make recommendations, further clinical trials with improved methodology are needed.

Caution is advised when considering adjuvant therapies for SSNHL due to the potential for harm and drug interactions with evidence-based treatments. Further research is required to determine the effectiveness and safety of these therapies in managing SSNHL.

Study limitations

It is important to acknowledge the limitations of our study. Firstly, the retrospective nature of the research design may have introduced biases and limited the availability of certain data. Additionally, the sample size was relatively small, which may impact the generalizability of the findings. Moreover, the study was conducted at a single tertiary hospital, which may limit the broader applicability of the results.

## Conclusions

Our study showed that adjuvant drugs were ineffective and may be unnecessary in the treatment. There is a likely connection between metabolic disorders and the presence of SSNHL. In our study, steroid treatment was the only therapeutic option that improved hearing recovery in diabetic patients. Additionally, healthcare professionals should prioritize early intervention due to being the main prognostic factor to maximize auditory recovery.
